# Chloral and Cerebral Disease

**Published:** 1871-05

**Authors:** 


Chloral and Cerebral Disease.—In the *Phila. Med. and Surg. Reporter*, a case is mentioned in which twenty-five grains of hydrate of chloral were taken to overcome a wakeful and nervous condition, with the effect of producing sleep, followed next morning by vertigo and disturbance of vision. A case occurred recently in this city, in which the same remedy was prescribed by a physician to a lady laboring under cerebral excitement. Under the influence of the chloral she became maniacal, and committed suicide.—*Pacific Medical Journal.*

